# Exploring Plant *α*‐Amylase Inhibitors: Mechanisms and Potential Application for Insect Pest Control

**DOI:** 10.1002/biot.70098

**Published:** 2025-08-19

**Authors:** Marcos Fernando Basso, Arnubio Valencia‐Jiménez, Fabrizio Lo Celso, Isabel Rodrigues Gerhardt, Thomas Joseph V. Higgins, Maria Fatima Grossi‐de‐Sa

**Affiliations:** ^1^ Embrapa Genetic Resources and Biotechnology Brasília Distrito Federal Brazil; ^2^ National Institute of Science and Technology, INCT PlantStress Biotech, EMBRAPA, Brasília, Distrito Federal Brazil; ^3^ Departamento De Producción Agropecuaria University of Caldas Manizales Caldas Colombia; ^4^ Department of Physics and Chemical University of Palermo Palermo Italy; ^5^ Embrapa Digital Agriculture Campinas São Paulo Brazil; ^6^ Genomics for Climate Change Research Center Campinas São Paulo Brazil; ^7^ Commonwealth Scientific and Industrial Research Organisation (CSIRO), Agriculture and Food Canberra Australia; ^8^ Catholic University of Brasília, Brasília Distrito Federal Brazil

**Keywords:** *α*‐amylase, crop protection, insecticidal protein, starch catabolism, transgenic plants

## Abstract

*α*‐Amylases are found in microbes, plants, and animals, including insect pests. They play crucial roles in catalyzing the hydrolysis of *α*‐1,4‐glucan bonds within starch, glycogen, and related carbohydrates, forming shorter oligomers. In green plants, these enzymes are pivotal for starch degradation during photosynthesis and seed germination, whereas in phytophagous insect pests, they predominantly facilitate seed parasitism by degrading raw starch granules. Amylase inhibitors in plants appear to function as part of their defense against pests and pathogens. In the context of insect pests, some of these amylase inhibitors can target *α*‐amylases in the digestive system of certain insects. Both mono‐ and dicotyledonous plants harbor multiple genes encoding proteinaceous *α*‐amylase inhibitors. Previous studies have demonstrated that *α*‐amylase inhibitors, whether produced in vitro or overexpressed in transgenic plants, can exhibit entomotoxic activity against certain insect pests. Field trials involving transgenic plants that overexpress *α*‐amylase inhibitors have been conducted, laying the foundation for the potential commercialization of crops engineered with these genes. Herein, this review explores the molecular interactions between plant *α*‐amylase inhibitors and insect *α*‐amylases, shedding light on the underlying mechanisms of action, structural diversity, and assessing the broader biotechnological applications of this promising strategy.

## Graphical Abstract and Lay Summary

In this study, we address the importance of α‐amylases in insect pests, the possible biochemical role of α‐amylase inhibitors in plant resistance to insect pests, the molecular relationships between plant α‐amylase inhibitors and insect α‐amylases, and the biotechnological potential of proteinaceous α‐amylase inhibitors for the control of insect pests.

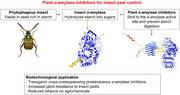

**Abbreviations**
AAI: Amaranth amylase inhibitor
Amyrels: *α*‐Amylase‐paralogs
BAS: Barley *α*‐amylase/subtilisin inhibitor
HbASI: *Hevea brasiliensis α*‐amylase/subtilisin inhibitor
ICK: Inhibitor cystine knots
LASI: *Ligusticum chuanxiong* subtilisin/*α*‐amylase inhibitor

## 1 Introduction

*α*‐Amylases are endo‐amylolytic enzymes classified as *α*‐1,4‐glucan‐4‐glucanohydrolases (EC 3.2.1.1), catalyzing the hydrolysis of *α*‐1,4‐glucosidic linkages within starch, glycogen, and related carbohydrates, yielding shorter mono‐ or oligomers [1, 2]. These enzymes belong to glycoside hydrolase family 13 (GH13; IPR013775) and are present in a wide range of organisms, including archaea, microorganisms, plants, insects, and other animals [3]. In plants, *α*‐amylases play a crucial role in breaking down reserve carbohydrates produced during photosynthesis into energy for cellular respiration. They are also essential for the degradation of starch granules during seed germination [4, 5, 6]. Phytophagous insects utilize *α*‐amylase to enhance their digestive capacity throughout developmental stages, particularly in starchy seed parasitism and raw starch granule degradation [7]. Mitigating insect‐mediated damage, green plants produce various proteinaceous and non‐proteinaceous molecules acting as *α*‐amylase inhibitors, thereby reducing plant or seed susceptibility to pathogens and insect pests [8, 9, 10].

The use of these inhibitory molecules has been proposed to enhance plant resistance to insect pests [11, 12, 13, 14]. Several coleopteran species, such as seed weevils and pod borers, pose significant threats to various crops, resulting in reduced seed quality and yield [15, 16, 17]. Consequently, several proteinaceous *α*‐amylase inhibitor genes from different plant species have been explored for transgenic overexpression in crops, which effectively enhances plant resistance to insect pests [18, 19, 20, 21, 22, 23, 24, 25, 26, 27]. Herein, we address the (i) importance of *α*‐amylases in insect pests, (ii) the possible biochemical role of *α*‐amylase inhibitors in plant resistance to insect pests, (iii) the molecular relationships between plant *α*‐amylase inhibitors and insect *α*‐amylases, and (iv) the biotechnological potential of proteinaceous *α*‐amylase inhibitors for the control of insect pests.

## 2 Insect‐Derived *α*‐Amylases

Insect *α*‐amylases belong to the GH13_15 subfamily and are characterized by a tripartite structure comprising domains A, B, and C, requiring calcium ions for enzymatic activity and sharing structural similarities with plant *α*‐amylases [28, 29, 30]. These enzymes are encoded by multiple genes that are expressed in a tissue‐specific or stage‐specific manner during insect development [3, 31, 32]. The number of genes varies among insect orders, ranging from 1 to 13 in dipterans (e.g., *Aedes albopictus*), 2 to 5 in lepidopterans (e.g., *Helicoverpa armigera*), 1 to 9 in coleopterans (e.g., *Tribolium castaneum*), 1 to 5 in hymenopterans (e.g., *Nasonia vitripennis*), and 0 to 5 genes in hemipterans (e.g., *Bemisia tabaci*) [29]. This abundance of genes may reflect the need for increased production of digestive enzymes to counteract the inhibitory effects of *α*‐amylase inhibitors [33]. Furthermore, the number of gene copies, expression profiles, tissue‐specific expression, and enzyme isoforms are positively correlated with the dietary habits of insects across generations [34, 35, 36, 37, 38]. An intriguing aspect of insect *α*‐amylases is how they interact with plant defense mechanisms, particularly *α*‐amylase inhibitors. Phytophagous insects are often confronted with plant‐produced molecules that interfere with their ability to feed, such as protein‐based inhibitors that are common in cereal and legume crops. At the molecular level, researchers have explored how insect *α*‐amylases interact with inhibitors to understand why similar enzymes show drastically different sensitivities. The three‐dimensional (3D) structure of the *α*‐amylase from *Tenebrio molitor* has been studied in combination with various inhibitors [28, 39]. Unlike mammalian *α*‐amylases, *T. molitor α*‐amylases lack a flexible loop, which was thought to explain the differences in sensitivity to inhibitors, particularly when compared to porcine *α*‐amylase, where the loop shifts in response to an inhibitor [40]. However, many insect pest *α*‐amylases have this loop, suggesting that it may not be the main factor determining sensitivity [41, 42]. The loops extending from inhibitors interact with the enzyme's catalytic site via ionic and hydrogen bonds and can block the sugar‐binding subsites [39]. This interaction depends on a large number of amino acids interface between the two proteins [43]. Furthermore, the efficacy of inhibitors depends on their ability to resist breakdown by insect gut proteases [43, 44]. Insect pest *α*‐amylases are primarily secreted via apocrine (lepidopteran and coleopteran) or exocytosis (dipterans) mechanisms in the midgut or foregut, with salivary accumulation occurring for potential extraoral activity [36, 45, 46, 47]. For instance, *Podisus maculiventris* and *Lygus lineolaris* utilize salivary *α*‐amylases for extraoral food digestion [38, 48], whereas carbohydrate digestion in *Zabrotes maculatus* and *Callosobruchus chinensis* primarily occurs in the midgut lumen [41]. The optimal pH for *α*‐amylase activity varies among insect groups, with coleopterans, hemipterans, and hymenopterans exhibiting optimal activity at acidic pH, dipterans at neutral pH, and lepidopterans at alkaline pH, corresponding to pH values in the midgut lumen [29, 45]. In addition, starch granules can have different structures and compositions, rendering them resistant to *α*‐amylases from certain insect groups and susceptible to others [49]. Non‐synonymous point mutations or minor structural changes can affect enzyme stability and substrate or inhibitor affinity, with chewing serving as a critical process in bruchids to disrupt starch granule structures and facilitate access to *α*‐amylase [50, 51]. Furthermore, *α*‐amylase‐related enzymes, called *α*‐amylase‐paralogs (Amyrels), classified within the GH13_15 subfamily, exhibit approximately 61% sequence identity and conserved functional domains resembling typical *α*‐amylases [3, 29, 30, 52, 53]. Although Amyrel enzymes display lower amylolytic activity compared to *α*‐amylases, they demonstrate both hydrolytic *α*‐amylase activity and 4‐*α*‐glucanotransferase transglycosylation activity, with optimal activity at neutral to slightly acidic pH [54]. Unlike *α*‐amylases, Amyrel enzymes can process maltotriose and maltose, yielding glucose and maltose as end products [54].

## 3 Plant‐Derived Proteinaceous *α*‐Amylase Inhibitors

Proteinaceous inhibitors are prevalent in microorganisms and plant vegetative tissues and seeds. They play a crucial role in controlling the activity of endogenous *α*‐amylases [13, 55] and provide defense against pathogens and insect pests [55, 56]. This group of inhibitors is often referred to as antimicrobial peptides, and some are recognized as antinutritional factors in plants or seeds with implications for human and animal nutrition [57]. These proteinaceous *α*‐amylase inhibitors can be grouped into seven distinct protein families based on similarities in sequence and 3D structures [57]. While several families exhibit bifunctional properties, inhibiting both *α*‐amylases and proteases, six are specific *α*‐amylase inhibitors [58, 59]. Some inhibitors exhibit high specificity for certain *α*‐amylases, while others display broader specificity across different *α*‐amylases [9]. The six families of plant proteinaceous *α*‐amylase inhibitors include knottins (or inhibitor cystine knots; ICKs), γ‐thionins (or defensins), CM‐proteins (or cereal‐type), kunitz‐type, thaumatin‐like, and lectin‐like [55, 60].

### 3.1 Knottins

Knottin family inhibitors are thermally and chemically ultra‐stable proteins rich in cysteine and proline. They commonly occur as 5–6 kDa proteins with 30–50 amino acid residues and also as conserved knottin domains in larger protein molecules (Table 1; Supporting Information File S1). In addition to plants, knottin *α*‐amylase inhibitors and proteins are found in various organisms, including animals, fungi, bacteria, and viruses. The Uniprot database reports 14,506 knottin‐related sequences organized into three distinct groups defined based on sequence alignment [61]. Group I comprises knottin inhibitors characterized by disulfide bonds between the knot‐forming cysteines III and VI, going through cystines I–IV and II–V [61]. Group II consists of cyclotide inhibitors with the same disulfide connectivity as knottins but with their backbone cyclized via an N‐terminal to C‐terminal peptide bond [61]. Group III includes the growth factor cystine‐knot inhibitors with different connectivity than knottins and cyclotides [61, 62]. A notable example is the AAI (or AhAI; amaranth amylase inhibitor) knottin inhibitor isolated from *Amaranthus hypochondriacus*. It contains a 26 amino acid signal peptide, followed by a 43 amino acid region with unknown function, 32 amino acid residues of the mature peptide, and three disulfide bonds [63]. The *AAI* gene exhibits higher expression in mature inflorescence, while the AAI inhibitor protein is active against *α*‐amylases of *T. castaneum* and *C. chinensis* [39, 63, 64, 65]. The AAI protein structure consists of a short triple‐stranded β‐sheet stabilized by three disulfide bonds, forming a typical knottin or inhibitor cystine knot fold essential for binding various macromolecular ligands [66, 67, 68]. The intercystine residues of the AAI inhibitor are crucial for binding to *α*‐amylase [66].

**TABLE 1:** In silico features of plant‐ and actinobacteria‐derived *α*‐amylase inhibitor proteins belonging to the seven families retrieved from the UniProt database.

Inhibitor family	Protein common name	UniProt code	Organism	Amino acid	MW (kDa)	Subcellular location	InterPro	CDD	Pfam	Gene Ontology	AlphaFold SWISS‐Model
Knottin ICK	Trypsin inhibitor 5	P83396 ITR5_CYCPE	*Cyclanthera pedata*	29	3.19	Secreted	IPR000737 IPR011052	cd00150	PF00299	GO:0005576 GO:0004867 GO:0030414 GO:0004866	P83396
	Trypsin inhibitor 2	P82409 ITR2_MOMCO	*Momordica cochinchinensis*	34	3.47	Secreted	IPR000737 IPR011052	cd00150	PF00299	GO:0005576 GO:0004867 GO:0030414 GO:0004866 GO:0006952 GO:1900004	P82409
	Trypsin inhibitor 3	P83394 ITR3_CYCPE	*Cyclanthera pedata*	29	3.19	Secreted	IPR000737 IPR011052	cd00150	PF00299	GO:0005576 GO:0004867 GO:0030414 GO:0004866	P83394
	Trypsin inhibitor 3	P10293 ITR3_CUCPE	*Cucurbita pepo*	32	3.65	Secreted	IPR000737 IPR011052	cd00150	PF00299	GO:0005576 GO:0004867 GO:0030414 GO:0004866	P10293
	Trypsin inhibitor 3	P32041 ITR3_CUCMC	*Cucumis melo*	30	3.40	Secreted	IPR000737 IPR011052	cd00150	PF00299	GO:0005576 GO:0004867 GO:0030414 GO:0004866	P32041
γ‐thionin defensin	Amylase inhibitor‐like protein	A9UIE0 A9UIE0_WHEAT	*Triticum aestivum*	82	8.1	No	IPR008176 IPR003614 IPR036574	No	PF00304	GO:0006952	A9UIE0
	Amylase inhibitor‐like protein	A9UID9 A9UID9_WHEAT	*Triticum aestivum*	82	8.83	No	IPR008176 IPR003614 IPR036574	No	PF00304	GO:0006952	A9UID9
	PDF1	Q8W434 Q8W434_VIGRA	*Vigna radiata*	75	8.63	No	IPR008176 IPR003614 IPR036574	No	PF00304	GO:0006952 GO:0031640 GO:0050832	Q8W434
	Defensin	B6VEV8 B6VEV8_VIGUN	*Vigna unguiculata*	47	5.41	No	IPR008176 IPR003614 IPR036574	No	PF00304	GO:0006952 GO:0050832 GO:0031640	B6VEV8
	Defensin‐like protein 1	Q7M1F2 DEF1_CLITE	*Clitoria ternatea*	49	5.61	Secreted	IPR003614 IPR036574	No	PF00304	GO:0006952 GO:0050832 GO:0031640 GO:0005576	Q7M1F2
CM‐protein Cereal‐type	Alpha‐amylase/trypsin inhibitor CMa	P28041 IAAA_HORVU	*Hordeum vulgare*	145	15.50	Endosperm	IPR006106 IPR006105 IPR036312 IPR016140	cd00261	PF00234	GO:0004867 GO:0015066 GO:0005576 GO:0030414	P28041
	CM 17 protein	Q41540 Q41540_WHEAT	*Triticum aestivum*	143	15.98	Secreted	IPR006106 IPR006105 IPR036312 IPR016140	cd00261	PF00234	GO:0004867 GO:0005576	Q41540
	Alpha‐amylase/trypsin inhibitor CM2	P16851 IAAC2_WHEAT	*Triticum aestivum*	145	15.46	Secreted Endosperm	IPR006106 IPR006105 IPR036312 IPR016140	cd00261	PF00234	GO:0004867 GO:0015066 GO:0005576 GO:0030414	P16851
	Alpha‐amylase/trypsin inhibitor CM1	P16850 IAAC1_WHEAT	*Triticum aestivum*	145	15.51	Secreted	IPR006106 IPR006105 IPR036312 IPR016140	cd00261	PF00234	GO:0004867 GO:0015066 GO:0005576 GO:0030414	P16850
	Alpha‐amylase/trypsin inhibitor CM16	P16159 IAC16_WHEAT	*Triticum aestivum*	143	15.78	Secreted	IPR006106 IPR006105 IPR036312 IPR016140	cd00261	PF00234	GO:0004867 GO:0015066 GO:0005576 GO:0030414	P16159
Kunitz‐type	Alpha‐amylase/subtilisin inhibitor	P07596 IAAS_HORVU	*Hordeum vulgare*	203	22.16	No	IPR011065 IPR002160	cd00178	PF00197	GO:0015066 GO:0004867 GO:0004866 GO:0030414	P07596
	Alpha‐amylase/subtilisin inhibitor	P29421 IAAS_ORYSJ	*Oryza sativa*	200	21.41	No	IPR011065 IPR002160	cd00178	PF00197	GO:0015066 GO:0004867 GO:0030414	P2942
	Endogenous alpha‐amylase/ subtilisin inhibitor	P16347 IAAS_WHEAT	*Triticum aestivum*	180	19.63	No	IPR011065 IPR002160	cd00178	PF00197	GO:0015066 GO:0004867 GO:0004866 GO:0030414	P16347
	Endogenous alpha‐amylase/ subtilisin inhibitor	W9RVA3 9RVA3_9ROSA	*Morus notabilis*	192	21.19	No	IPR011065 IPR002160	No	PF00197	GO:0004866 GO:0008233 GO:0006508	W9RVA3
	Alpha amylase inhibitor	E6Y8T1 E6Y8T1_ORYSI	*Oryza sativa*	146	15.67	No	IPR011065 IPR002160	cd00178	PF00197	GO:0004866	E6Y8T1
Thaumatin‐like	Zeamatin‐like protein	B8QXV0 B8QXV0_ZEAMP	*Zea mays*	228	24.08	No	IPR037176 IPR001938 IPR017949	No	PF00314	GO:0006952 GO:0050832 GO:0031640	B8QXV0
	Alpha‐amylase/trypsin inhibitor	P13867 IAAT_MAIZE	*Zea mays*	206	22.07	No	IPR037176 IPR001938 IPR017949	No	PF00314	GO:0006952 GO:0050832 GO:0030414 GO:0031640 GO:0004867	P13867
	Zeamatin‐like protein	C5XCE2 5XCE2_SORBI	*Sorghum bicolor*	233	24.56	No	IPR037176 IPR001938 IPR017949	No	PF00314	GO:0006952	C5XCE2
	Zeamatin	P33679 ZEAM_MAIZE	*Zea mays*	227	23.99	No	IPR037176 IPR001938 IPR017949	No	PF00314	GO:0006952 GO:0050832 GO:0031640	P33679
	Alpha‐amylase/trypsin inhibitor, putative, expressed	Q2QLS3 Q2QLS3_ORYSJ	*Oryza sativa*	258	26.52	No	IPR037176 IPR001938 IPR017949	No	PF00314	GO:0006952	Q2QLS3
Lectin‐like	Alpha‐amylase inhibitor 1	P02873 LEA1_PHAVU	*Phaseolus vulgaris*	246	27.20	No	IPR013320 IPR016363 IPR000985 IPR019825 IPR001220	cd06899	PF00139	GO:0030246 GO:0015066 GO:0030246	P02873
	Alpha‐amylase inhibitor 2	Q41114 LEA2_PHAVU	*Phaseolus vulgaris*	240	26.59	No	IPR013320 IPR016363 IPR000985 IPR019825 IPR001220	cd06899	PF00139	GO:0030246 GO:0015066 GO:0030246	Q41114
	Alpha‐amylase inhibitor	Q43630 Q43630_PHAVU	*Phaseolus vulgaris*	262	28.98	No	IPR013320 IPR016363 IPR000985 IPR001220	cd06899	PF00139	GO:0030246 GO:0015066 GO:0030246	Q43630
	Alpha amylase inhibitor	A9 × 5V1 A9 × 5V1_LABPU	*Lablab purpureus*	274	29.93	No	IPR013320 IPR016363 IPR000985 IPR001220	cd06899	PF00139	GO:0030246	A9 × 5V1
	Alpha‐amylase inhibitor	Q5ZEW6 Q5ZEW6_9FABA	*Phaseolus costaricensis*	244	26.96	No	IPR013320 IPR016363 IPR000985 IPR019825 IPR001220	cd06899	PF00139	GO:0030246 GO:0015066 GO:0030246	Q5ZEW6
Actinobacteria	Alpha‐amylase inhibitor Z‐2685	P07512 IAA_STRRO	*Streptomyces rochei*	76	8.13	No	IPR000833 IPR036379	No	PF01356	GO:0015066	P07512
	Alpha‐amylase inhibitor Paim‐2	P20596 IAA2_STROI	*Streptomyces olivaceoviridis*	75	7.62	No	IPR000833 IPR036379	No	PF01356	GO:0015066	P20596
	Alpha‐amylase inhibitor AI‐3688	P04082 IAA_KITAU	*Kitasatospora aureofaciens*	36	3.93	No	IPR000833 IPR036379	No	PF01356	GO:0015066	P04082
	Alpha‐amylase inhibitor Haim‐1	P01093 IAA1_STRGS	*Streptomyces griseosporeus*	78	8.12	No	IPR000833 IPR036379	No	PF01356	GO:0015066	P01093
	Alpha‐amylase inhibitor	A0A3S8Y247 A0A3S8Y247_9ACTN	*Streptomyces* sp.	109	11.32	No	IPR000833 IPR036379	No	PF01356	GO:0015066 GO:0016829	A0A3S8Y247

*Note*: UniProt database [111]; Pfam database [141]; CDD webserver [142]; InterPro database [143].

### 3.2 γ‐Thionins

γ‐Thionin family inhibitors are small and stable plant proteins that are 45–55 amino acids long and are sulfur amino acid‐rich. They play a role in plant defense by strongly inhibiting insect pest *α*‐amylases and proteases (Table 1; Supporting Information File S2) [69, 70, 71, 72]. These inhibitors are found exclusively in plants, with 5803 thionin‐related sequences reported in the UniProt database. They are classified into a superfamily and organized into four major groups based on their mode of action, primary and secondary structure, and disulfide bond pattern [73]. Groups I and II are known for their toxicity to bacteria and fungi. In contrast, groups III and IV exhibit extreme cytotoxicity to mammals [73], with several that can be toxic to insect pests [74, 75]. The 3D structure of these γ‐thionins is characterized by a typical two‐layer αβ sandwich that creates an amphipathic molecule with highly conserved cysteine residues [71, 76, 77]. Certain γ‐thionins can inhibit digestive enzymes, placing them among a unique class of plant proteins with the ability to block insect *α*‐amylases [70]. A proposed inhibitory mechanism suggests that γ‐thionin forms a Ca^2+^/inhibitor complex on membranes, leading to the chelation of calcium ions and destabilization of calcium‐dependent *α*‐amylases [73, 78, 79].

### 3.3 CM‐Proteins

The CM‐protein family (chloroform‐methanol soluble) of inhibitors (Table 1) consists of versatile proteins, typically comprising mono‐ or bifunctional entities of 120–160 amino acids. These proteins are commonly present in aqueous extracts of cereal and grass seeds, constituting 2%–4% of the total protein content [80]. These inhibitors contain four to five conserved disulfide bonds, rendering them highly stable and multi‐dimeric. Known for their sulfur amino acid‐rich composition, they exhibit inhibitory activity against insect pests and mammalian *α*‐amylases (Table 1; Supporting Information File S3) [81, 82]. In addition to their prevalence in plants, CM‐proteins also exist in microorganisms as homologous proteins and are categorized in the prolamine superfamily. The UniProt database reports 795 CM‐protein‐related sequences, which are organized into five major groups: mono/dimeric, heterodimeric, LDI‐like, protease inhibitors, and trypsin inhibitors. Structurally, CM‐proteins adopt a four‐helix bundle configuration and form enzyme complexes capable of binding versatility, involving various regions, including the N‐terminal and different loop regions [80].

### 3.4 Kunitz‐Type

The kunitz‐type of plant‐derived *α*‐amylase inhibitors are characterized by low molecular weight bifunctional proteins, typically comprising 180 amino acids. With an approximate molecular weight of 18–24 kDa, these inhibitors are organized into one or two polypeptide chains linked by one or two disulfide bonds. With up to five cysteine residues, they adopt a β trefoil topology (Table 1; Supporting Information File S4) [83, 84, 85]. While prevalent in plants, kunitz‐type inhibitors and proteins are also found in animals, evidenced by 34,515 kunitz‐related sequences in the UniProt database. Among the well‐studied plant‐derived kunitz‐type inhibitors are *Ligusticum chuanxiong* subtilisin/*α*‐amylase inhibitor (LASI), barley *α*‐amylase/subtilisin inhibitor (BASI), and *Hevea brasiliensis α*‐amylase/subtilisin inhibitor (HbASI). These inhibitors demonstrate inhibitory effects against mammalian, fungal, and insect pest *α*‐amylases [86, 87, 88]. For instance, the BASI inhibitor exhibits strong interaction with both A and B domains of the AMY2 *α*‐amylase near the catalytic site, mediated by hydrogen bonds, salt bridges, and van der Waals interactions, effectively impeding substrate access [9].

### 3.5 Thaumatin‐Like

The thaumatin‐like family of inhibitors is composed of bifunctional proteins consisting of 173 to 235 amino acids. Their molecular weights range from 17 to 26 kDa and feature 10 to 16 conserved cysteine residues that can form five to eight disulfide bonds. They exhibit high sequence similarities to the pathogenesis‐related 5 (PR‐5) protein family (Table 1; Supporting Information File S5) [89, 90]. Thaumatin‐like inhibitors are found in both plants and microorganisms, with 19,586 thaumatin‐related sequences reported in the UniProt database. Encoded by numerous genes commonly up‐regulated in response to various stresses, the inhibitors mainly possess antifungal activity [91]. However, zeamatin, a thaumatin‐like protein isolated from maize, exhibits significant inhibitory effects against insect pest *α*‐amylase and trypsin enzymes [9, 89]. In the same way, as the Pathogenesis‐related (PR) protein PR‐5d comprises an acidic cleft in its amino acid structure [92], the NP24‐I isoform of the thaumatin‐like protein NP24 isolated from tomato also displays a distinctive structure characterized by a prominent acidic cleft between domains I and II [90].

### 3.6 Lectin‐Like

The lectin‐like family of inhibitors is 30–50 kDa bifunctional proteins comprising 240–250 amino acid residues (Table 1; Supporting Information File S6) [12, 93, 94, 95, 96]. Notable examples include *α*AI‐1, *α*AI‐2, *α*AI‐3, and *α*AI‐0 identified in common bean seeds [9, 97]. While predominantly found in plants, lectin‐like inhibitors are also found in animals and microorganisms, with 12,806 lectin‐related sequences in the UniProt database. For instance, *α*AI‐1 isolated from common or kidney beans inhibits both mammalian and insect pest *α*‐amylases by mimicking the substrate and interacting directly with catalytic residues via hydrogen bonds [55, 98, 99, 100]. The proteolytic processing of homodimers at an Asn‐Ser site is crucial for acquiring the *α*2β2 subunit composition necessary for the activation of *α*AI‐1 inhibitory activity [97, 101]. Although it shares high sequence homology with *α*AI‐1, *α*AI‐2 has significant differences in its glycosylation pattern and a critical binding region between the enzyme and inhibitor [102, 103]. It was also less effective than *α*AI‐1 in protecting peas from *Bruchus pisorum* in the field [20]. The *α*AI‐1 inhibits pea bruchid α‐amylase by 80% within a pH range of 4.5–6.5, while *α*AI‐2 is significantly less effective, reducing enzyme activity by only 40% at pH 4.0–4.5. Furthermore, *α*AI‐1 in pea seeds can provide complete protection against *B. pisorum*, leading to larval mortality at the first or second instar. In contrast, *α*AI‐2 appears to cause a delay in larval maturation [20].

Insect pests use several hydrolytic enzymes, including *α*‐amylases, either within or secreted through the digestive tract. These enzymes help to break down barriers and make starchy food available when the pests parasitize plants or their seeds. The expression of genes encoding hydrolytic enzymes is modulated, depending on the diet and the different life stages of the insect pest [32, 41]. Co‐evolving with starchy plant foods, these insect pests have mechanisms to evade or cope with plant inhibitors [8]. Conversely, plants possess reservoirs, such as protein storage vacuolar bodies. While some plant species can induce the production of defensive proteins in response to herbivore damage, such inducible accumulation of *α*‐amylase inhibitors in seeds has not been conclusively demonstrated [9, 93]. Alternatively, inhibitors may be pre‐accumulated in cereal and legume seeds to varying degrees depending on plant species and genotype [9]. For example, a lectin‐related *α*AI is synthesized as isoforms in the endoplasmic reticulum, *N‐*glycosylated, and transported via the secretory pathway from Golgi‐derived vesicles. It accumulates in the protein storage vacuole of cotyledonary cells during seed development [104]. Within the vacuole, the *α*AI undergoes processing involving the removal of specific amino acid residues from the C‐terminus and subsequent endoproteolytic cleavage, resulting in various active forms [104, 105]. Generally, plant‐derived proteinaceous inhibitors act by binding to the specific active or catalytic site of the target insect enzyme [9, 14, 51]. However, other non‐competitive biochemical changes in the target enzyme may also occur due to inhibitor action. The specific amino acid residues in the contact region between *α*‐amylases and their inhibitors, or alterations in multi‐structural properties (e.g., steric, electrostatic, hydrogen bond, conformational, and disulfide bond), are considered determinants for inhibitor specificity [51, 105, 106]. Furthermore, the substrate‐binding pocket contributes to the *α*‐amylases’ specificities, and loop variability near the enzyme active site determines the inhibitor specificity of *α*‐amylases. Conversely, specificity and binding affinities could be influenced by the conformation of the inhibitor [107]. The pH and temperature play a critical role in the formation of the enzyme/inhibitor complex and in determining inhibition efficiency, with acidic pH generally showing the highest activity [108, 109]. Additionally, factors such as exposure time and the concentration balance of both enzyme and inhibitor are pivotal [110].

## 4 Evolutionary Relationships Among Proteinaceous *α*‐Amylase Inhibitors

**FIGURE 1:**
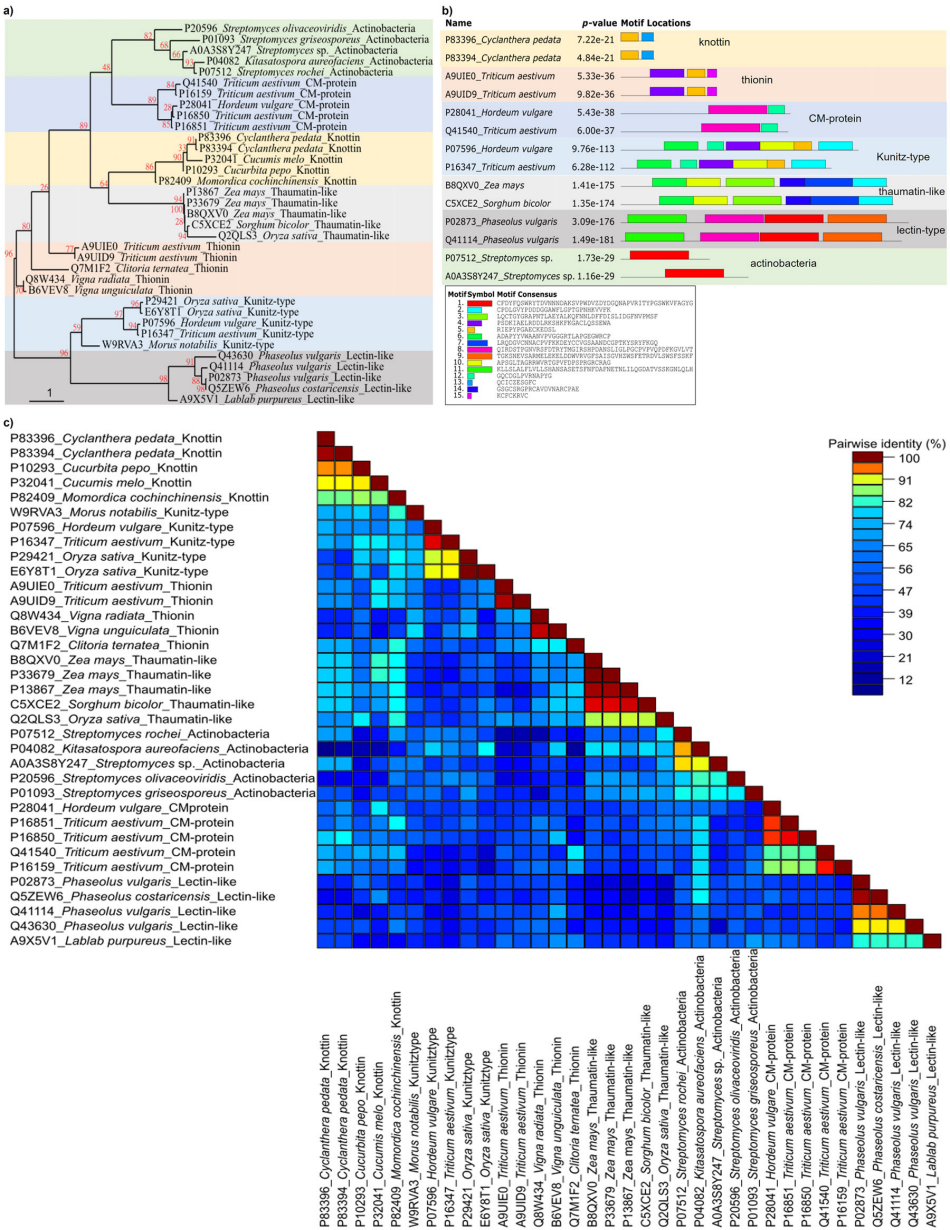
In silico analyses of the five more representative sequences of plant‐ and actinobacteria‐derived *α*‐amylase inhibitor proteins belonging to the seven families: knottin (or inhibitor cystine knot; ICK), γ‐thionin (or defensin), CM‐protein (or cereal‐type), kunitz‐type, thaumatin‐like, lectin‐like, and actinobacteria (*Streptomyces* sp.) (Table 1; Supporting Information Files S1 to S8), retrieved from UniProt database Release 2023_05 [111]. (A) The evolutionary tree was constructed from amino acid protein sequences (Supporting Information File S8) with the maximum likelihood method, Approximate Likelihood‐Ratio Test (aLRT) SH‐like branch support, and WAG substitution model using the Phylogeny.fr tool webserver [138]. (B) Conserved motifs in amino acid sequences (Supporting Information File S9) were identified using the MEME suite web server with default parameters [139]. (C) Pairwise identity matrix was generated from amino acid sequences (Supporting Information File S8) using the Sequence Demarcation Tool version 1.2 with default parameters [140].

A substantial number of *α*‐amylase inhibitor protein sequences from seven families are readily accessible in the UniProt database [111]. Currently, the database contains approximately 92 knottin/ICK sequences (Supporting Information File S1), seven γ‐thionin/defensin sequences (Supporting Information File S2), 686 CM‐proteins/cereal‐type sequences (Supporting Information File S3), 173 kunitz‐type sequences (Supporting Information File S4), 41 thaumatin‐like sequences (Supporting Information File S5), 78 lectin‐like sequences (Supporting Information File S6), and 459 actinobacteria sequences (Supporting Information File S7) of proteinaceous *α*‐amylase inhibitors derived from plants and actinobacteria, specifically from the *Streptomyces* genus. Each of these seven families of *α*‐amylase inhibitors exhibits high conservation of functional domains, with noteworthy domains including PF00299 for knottin/ICK, PF00304 for γ‐thionin/defensin, PF00234 for CM‐protein/cereal‐like, PF00197 for kunitz‐like, PF00314 for thaumatin‐like, PF00139 for lectin‐like, and PF01356 for actinobacteria (Table 1). Phylogenetic relationships within each inhibitor family are also conserved (Figure 1A). Additionally, the conserved motifs among the protein sequences are differential features among these seven families, although some are shared among different families (Figure 1B). High intra‐family pair‐to‐pair sequence identities were also observed (Figure 1C).

**FIGURE 2:**
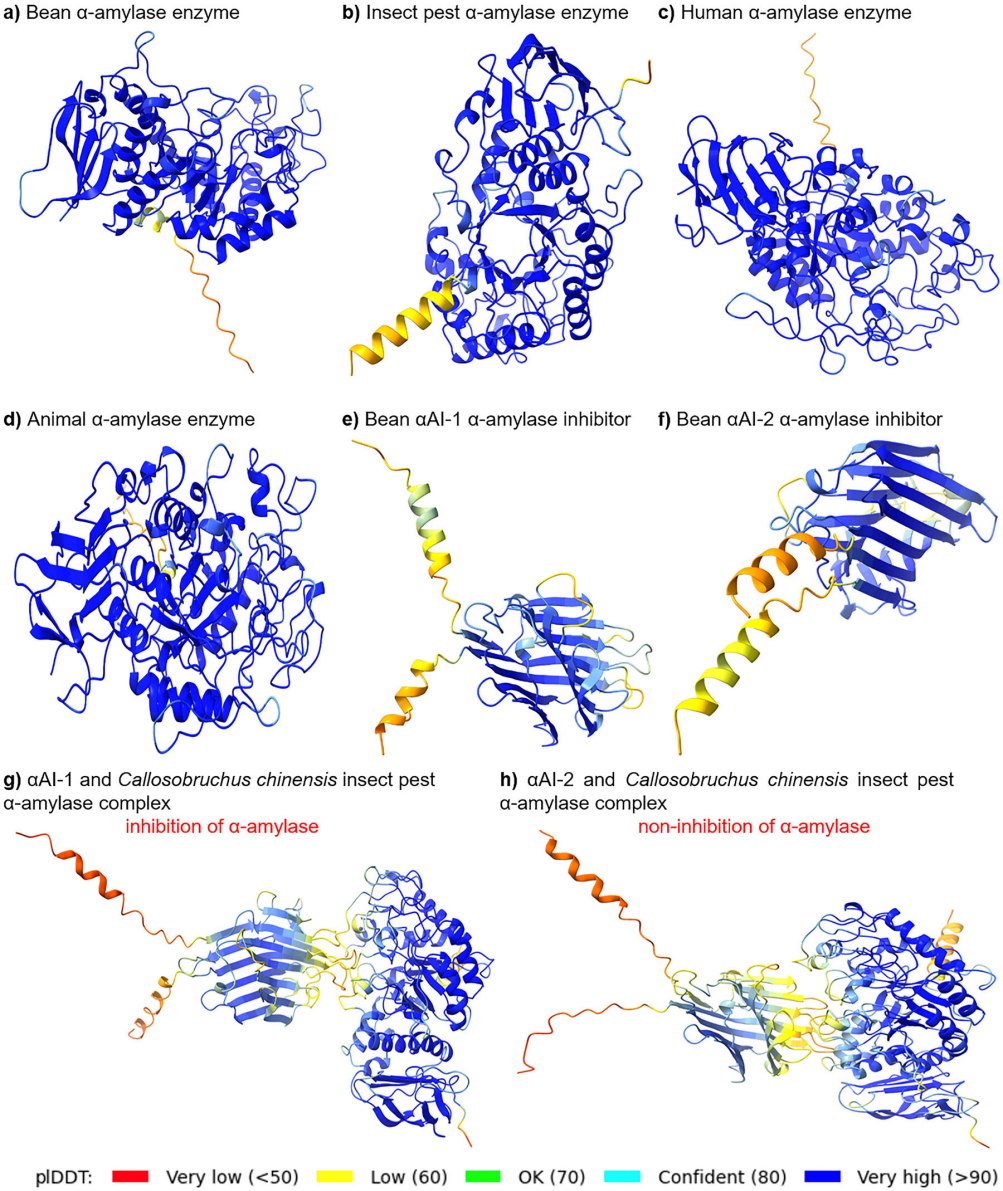
Representative three‐dimensional (3D) structures of different *α*‐amylases and plant *α*‐amylase inhibitors performed with AlphaFold2 and AlphaFold2‐multimer [112]. (A) 3D structure of a plant *α*‐amylase (*Phaseolus vulgaris*, V7CTK1_PHAVU). (B) 3D structure of an insect pest *α*‐amylase (*Callosobruchus chinensis α*‐amylase, A0A168VCK5_CALCS). (C) 3D structure of a human *α*‐amylase (Human pancreatic *α*‐amylase, P04746 AMYP_HUMAN). (D) 3D structure of an animal *α*‐amylase (Porcine pancreatic *α*‐amylase, P00690 AMYP_PIG). (E) 3D structure of a proteinaceous *α*‐amylase inhibitor (*P. vulgaris α*‐amylase inhibitor isoform 1, *α*AI‐1, P02873 LEA1_PHAVU). (F) 3D structure of a proteinaceous *α*‐amylase inhibitor (*P. vulgaris α*‐amylase inhibitor isoform 2, *α*AI‐2, Q41114 LEA2_PHAVU). (G) 3D structure of protein‐protein interaction between the plant‐derived proteinaceous *α*AI‐1 *α*‐amylase inhibitor (P02873 LEA1_PHAVU) and insect pest *α*‐amylase (*C. chinensis* α‐amylase, A0A168VCK5_CALCS). (H) 3D structure of protein‐protein interaction between the plant‐derived proteinaceous *α*AI‐2 *α*‐amylase inhibitor (Q41114 LEA2_PHAVU) and insect pest *α*‐amylase (*C. chinensis α*‐amylase, A0A168VCK5_CALCS). Importantly, biochemical studies showed that *P. vulgaris α*AI‐1 inhibits porcine pancreatic *α*‐amylase (PPA) and *α*‐amylases of *Callosobruchus maculatus* and *C. chinensis*, but does not inhibit *α*‐amylase of *Zabrotes subfasciatus*. In contrast, *P. vulgaris α*AI‐2 does not inhibit PPA and *α*‐amylases of *C. maculatus* and *C. chinensis*, but inhibits *α*‐amylase of *Z. subfasciatus*. Protein sequences were retrieved from the Uniprot database Release 2023_05 [111]. Regions of different degrees of confidence are expressed with different colors according to the predicted local distance difference test (plDDT) value.

**FIGURE 3:**
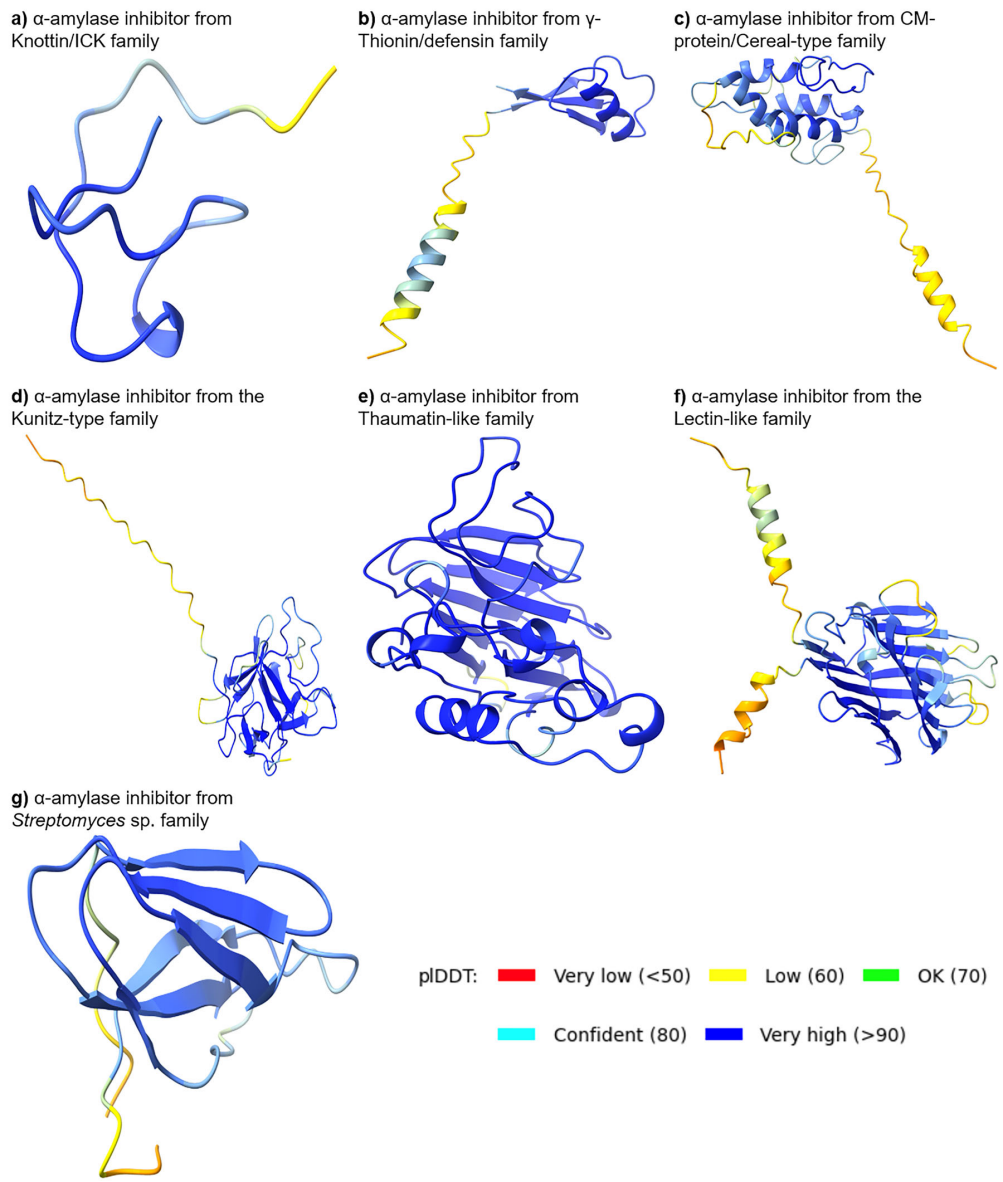
Representative three‐dimensional (3D) structures of different plant *α*‐amylase inhibitor proteins performed with AlphaFold2 and AlphaFold2‐multimer [112]. (A) 3D structure of a plant *α*‐amylase inhibitor from knottin/ICK family (*Cucumis melo*, P32041 ITR3_CUCMC). (B) 3D structure of a plant *α*‐amylase inhibitor from γ‐thionin/defensin family (*Vigna radiata*, Q8W434_VIGRA). (C) 3D structure of a plant *α*‐amylase inhibitor from CM‐protein/cereal‐type family (*Triticum aestivum*, P16851 IAAC2_WHEAT). (D) 3D structure of a plant *α*‐amylase inhibitor from the kunitz‐type family (*Hordeum vulgare*, P07596 IAAS_HORVU). (E) 3D structure of a plant *α*‐amylase inhibitor from the thaumatin‐like family (*Zea mays*, P13867 IAAT_MAIZE). (F) 3D structure of a plant *α*‐amylase inhibitor from the lectin‐like family (*Phaseolus vulgaris*, P02873 LEA1_PHAVU). (G) 3D structure of an actinobacteria *α*‐amylase inhibitor from the actinobacteria family (*Streptomyces rochei*, P07512 IAA_STRRO). Protein sequences were retrieved from the Uniprot database Release 2023_05 [111]. Regions of different degrees of confidence are expressed with different colors according to the predicted local distance difference test (plDDT) value.

The 3D structures of *α*‐amylase performed with AlphaFold2 and AlphaFold2‐multimer [112] from bean (Figure 2A), insect (Figure 2B), human (Figure 2C), and porcine (Figure 2D) species, as well as the bean *α*‐amylase inhibitor isoform 1 (*α*AI‐1; Figure 2E) and isoform 2 (*α*AI‐2; Figure 2F), reveal structural conservation among *α*‐amylases and inhibitors. Furthermore, protein‐protein interaction complexes were elucidated between *α*AI‐1 and *α*AI‐2 versus the *C. chinensis* insect pest *α*‐amylase (Figure 2G,H). Similarly, the protein structures of *α*‐amylase inhibitors from the knottin/ICK (Figure 3A), γ‐thionin (Figure 3B), CM‐protein/cereal‐like (Figure 3C), kunitz‐type (Figure 3D), thaumatin‐like/defensin (Figure 3E), lectin‐like (Figure 3F), and actinobacteria (Figure 3G) families were provided.

## 5 Engineered *α*‐Amylase Inhibitors for Targeted Pest Control

Artificial *α*‐amylase inhibitors have been developed using various approaches. These include utilizing the CBD‐WT domain followed by an in vitro screening based on phage display [113], employing DNA shuffling and phage display techniques to generate combinatorial libraries containing inhibitor variant forms [26], and incorporating specific modifications to the PAMI (peptide amylase inhibitor) motif followed by screening in L‐peptide libraries [114]. Site‐directed mutations in *α*AI‐2 inhibitors have also been explored, identifying essential amino acids crucial for inhibitory activity, which suggests the potential for enhancing inhibitory activity through engineered mutations [14]. Silva et al. [26] identified three mutant *α*‐amylase inhibitors (*α*‐AIC3, *α*‐AIA4, and *α*‐AIG11) with remarkably high inhibitory activity against *Anthonomus grandis* (Coleoptera: Curculionidae) *α*‐amylase. These findings represent a significant advancement in insect pest management and agricultural biotechnology. This study elucidated the potential of engineered *α*‐amylase inhibitors to effectively control agricultural pests, thereby reducing crop damage and improving yield. The transient overexpression of the *α‐AIC3* mutant gene in *Nicotiana benthamiana* was investigated by Prado et al. [115], revealing promising results regarding *α‐*AIC3 production efficiency and inhibitory efficacy against *A. grandis α*‐amylases. This transient expression system demonstrated effectiveness in producing the mutant inhibitor, highlighting its potential for large‐scale production and practical application in pest management strategies. These findings underscore the importance of biotechnological approaches in developing novel solutions for agricultural challenges, particularly in combating insect pests that pose significant threats to crop productivity. By harnessing the power of genetic engineering and protein design, researchers may be able to tailor *α*‐amylase inhibitors to target specific pests while minimizing ecological impact and ensuring sustainable agricultural practices.

## 6 Biotechnological Tools Using Proteinaceous *α*‐Amylase Inhibitors in Crops for Insect Pest Control

Numerous plant species naturally accumulate varying levels of *α*‐amylase inhibitors that appear to serve as a defense mechanism against insect pests, particularly in legume and cereal seeds [116, 117]. This accumulation tends to be higher in wild accessions or progenitors compared to domesticated commercial varieties and is negatively correlated with the susceptibility of modern cereals to insect pests [106]. Biotechnological tools, such as the transgenic overexpression of genes encoding plant‐derived proteinaceous *α*‐amylase inhibitors, have been proposed for controlling various insect pests, notably bruchids in legume grains and weevils in cereals and other crops (Table 2) [18, 23, 44, 118, 119]. However, these are still at the research stage and have not yet reached commercial use. For example, weevil‐resistant field peas were not commercialized because peas can be part of stock feed diets that are not heat‐treated and may result in reduced feed efficiency in broiler chickens [120]. There are also a small number of granted and active patents involving the use of *α*‐amylase inhibitors to control insect pests (Table 3). Bruchids, in particular, have been a primary target of these technologies because of the significant damage they cause, especially to grain stored for long periods, where they consume starch and reproduce rapidly [20, 102]. Infestation by some of these insects can occur even during seed development in pods, persisting through storage and even during trade [8]. Furthermore, *α*‐amylase inhibitors have demonstrated efficacy against other coleopteran insects, such as cotton flower bud parasites (*A. grandis*) and coffee fruit pests (*Hypothenemus hampei*; Coleoptera: Curculionidae) [14, 22, 24, 26].

**TABLE 2:** Overview of transgenic plants overexpressing different genes encoding *α*‐amylase inhibitors to improve plant resistance against insect pests.

Inhibitor	Biotechnological strategy	Species	Insect target	Strategy	Results	Reference
Bean *α*‐amylase inhibitor 1 and 2 (*α*AI‐1 and *α*AI‐2)^a^	Transgenic overexpression	*Pisum sativum*	*Bruchus pisorum*	cDNA overexpression was driven by a phytohemagglutinin promoter. Transgenic seeds showed accumulation of up to 3% of soluble protein	The *α*AI‐1 inhibited pea bruchid *α*‐amylase by 80% over a broad pH range and caused larval mortality at the first or second instar in the lab and in the field. Meanwhile, *α*AI‐2 inhibited the *α*‐amylase enzyme by only 40%, and only in the pH 4.0–4.5 range, causing a delay in the maturation of the larvae under the field conditions	[19, 20]
Bean *α*‐amylase inhibitor 1 (*α*AI‐1)	Transgenic overexpression	*Coffea arabica*	*Hypotheneumus hampei*	cDNA overexpression was driven by a phytohemagglutinin promoter. Transgenic seeds showed accumulation of up to 0.29% protein in crude extracts	In vitro inhibitory assays of the *α*AI‐1 inhibitor against *H. hampei α*‐amylases using transgenic seed extracts showed up to 88% inhibition of *α*‐amylase enzyme activity	[24]
Bean *α*‐amylase inhibitor 2 (*α*AI‐2)	Transgenic overexpression	*Nicotiana tabacum*	*Zabrotes subfasciatus*	cDNA overexpression was driven by a phytohemagglutinin promoter	Unfortunately, all *α*AI‐2 mutant inhibitors lost their activity against the insect pest *α*‐amylases but none exhibited activity against mammalian *α*‐amylases	[14]
Bean *α*‐amylase inhibitor 1 (αAI‐1)	Transgenic overexpression	*Pisum sativum* *Vigna angularis*	*Zabrotes subfasciatus*	cDNA overexpression was driven by a phytohemagglutinin promoter. Transgenic seeds showed accumulation of up to 3% of soluble proteins	Transgenic seeds were resistant to the *Z. subfasciatus* whose insect *α*‐amylases are inhibited by *α*Al‐1	[8, 95]
Bean *α*‐amylase inhibitor (*α*AI‐Pv)	Transgenic overexpression	*Pisum sativum*	*Callosobruchus chinensis* *Callosobruchus maculatus*	cDNA overexpression was driven by a phytohemagglutinin promoter. Transgenic T_2_ seeds showed *α*AI‐Pv accumulation of up to 1.27% (weight/weight)	Transgenic pea seeds were resistant to *C. chinensis* and *C. maculates*. The overexpression of *α*AI‐Pv resulted in structurally modified forms of this inhibitor, which were initially considered more allergenic to mice than the original protein from beans, but this was later corrected when it was found that the bean form was, in fact, allergenic	[18, 122, 123, 124]
Bean *α*‐amylase inhibitor (*α*AI)	Transgenic overexpression	*Vigna angularis*	*Callosobruchus chinensis*, *C. maculatus*, and *Callosobruchus analis*	cDNA overexpression was driven by a phytohemagglutinin promoter. Transgenic *α*AI concentrations were 0.82% in transgenic seeds	Transgenic seeds showed a complete block of development and the emergence of both three bruchid species on these seeds	[44]
Wheat WMAI‐l *α*‐amylase inhibitor	Transgenic overexpression	*Nicotiana tabacum*	*Agrotis ipsilon*	cDNA overexpression was driven by the CaMV 35S promoter	Significant mortality of first‐instar larvae of *A. ipsilon* was observed when fed on transgenic tobacco. Larval mortality was 30%–40% higher than in non‐transgenic control plants	[144]
Corn bifunctional 14K‐CI *α*‐amylase inhibitor	Transgenic overexpression	*Nicotiana tabacum*	−	cDNA overexpression was driven by the CaMV 35S promoter. The inhibitor was expressed in amounts up to 0.05% of the total protein in young leaves	Low accumulation of 14K‐CI in transgenic plants. Protein extracts from transgenic plants were more inhibitory than non‐transgenic control plant extracts to bovine trypsin activity. However, insect pest susceptibility was not determined	[145]
Wheat WAI *α*‐amylase inhibitor	Transgenic overexpression	*Solanum tuberosum*	*Myzus persicae*	cDNA overexpression was driven by the CaMV 35S promoter. High levels of *WAI* mRNA were detected in some of the transformants, but WAI protein could not be detected	Transgenic plants overexpressing the *WAI* and *GNA* (snowdrop lectin) pyramided genes showed reduced plant susceptibility to *M. persicae* as a consequence of reduced insect fecundity, supported by 15%–20% delay in nymphal production	[145, 146]
Bean *α*AI‐1 and *α*AI‐2 *α*‐amylase inhibitor variant forms	Transgenic overexpression	*Arabidopsis thaliana*	*Anthonomus grandis*	cDNA overexpression was driven by the CaMV 2 × 35S promoter. The inhibitors were expressed in amounts between 0.2 and 0.3% of the leaf protein extract	Transgenic plant extracts of three *α*AI‐1/2 mutants, named *α*‐AIC3, *α*‐AIA11, and *α*‐AIG4 showed high inhibitory activities against *A. grandis α*‐amylases in an in vitro assay. The inhibition rate was 77% for α‐AIC3, 57% for α‐AIA11, and 74% for α‐AIG4 protein, while no inhibitory activity was observed with original α‐AI1	[26]
Bean *α*‐amylase inhibitor 2 (*α*AI‐2)	Transgenic overexpression	*Nicotiana tabacum*	*Zabrotes subfasciatus*	cDNA overexpression was driven by a phytohemagglutinin promoter	Transgenic seeds showed inhibitory activity against *Z. subfasciatus α*‐amylase but not against porcine pancreatic *α*‐amylase	[42]
Bean *α*‐amylase inhibitor 1 (*α*AI‐1)	Transgenic overexpression with a cotyledon‐specific promoter	*Cicer arietinum* *Vigna unguiculata*	*Acanthoscelides obtectus*, *Callosobruchus chinensis*, and *C. maculatus*	cDNA overexpression was driven by a phytohemagglutinin promoter. Transgenic seeds accumulated up to 4.2% of seed protein	All transgenic lines were highly resistant to both *Callosobruchus* species, while *A. obtectus*, known to be tolerant to *α*AI‐1 inhibitor, was able to develop in all transgenic lines. The emergence of adult *C. maculatus* and *C. chinensis* was reduced by more than 93%. No negative effects under non‐target hymenopteran bruchid parasitoids were observed	[21, 25, 147]
Bean *α*‐amylase inhibitor 1 (*α*AI‐1)	Transgenic overexpression	*Cicer arietinum*	*Callosobruchus maculatus*	cDNA overexpression was driven by a phytohemagglutinin promoter. Transgenic seeds accumulated up to 0.72% of seed protein	A significant reduction in the emergence, adult insect weight, adult longevity, and survival rate of *C. maculatus* was observed when fed on transgenic chickpea seeds, ranging from 66.5 to 80.7% mortality	[148]
Bean *α*‐amylase inhibitor 1 (*α*AI‐1)	Transgenic overexpression	*Vigna unguiculata*	*Callosobruchus maculatus* and *C. chinensis*	cDNA overexpression was driven by a phytohemagglutinin promoter	Transgenic plants overexpressing *α*AI‐1 strongly inhibited the development of *C. maculatus* and *C. chinensis* in insect bioassays, ranging from 64%–71% and 79%–82% mortality, respectively. Transgenic plants showed agronomic performance and phenotypic characteristics very similar to its non‐transgenic parental counterpart	[23, 27]
Rice RAG2 *α*‐amylase/trypsin inhibitor^$^	Transgenic overexpression	*Oryza sativa*	−	cDNA overexpression was driven by a ubiquitin 1 promoter	Transgenic plants showed an increased grain size and improved grain quality and yield simultaneously. RAG2 might play an important role in regulating grain weight and seed quality of rice. However, it has not been evaluated against insect pest	[149]
*Zea mays* defensin 1 (ZmDEF1)	Transgenic overexpression	*Zea mays*	*Sitophilus zeamais*	cDNA overexpression was driven by a phaseolin promoter	The recombinant ZmDEF1 protein inhibited the *α*‐amylase activity of *S. zeamais* larvae at 54%–63% greater than the ZmDEF1 protein extracted from non‐transgenic plants	[150]
*Secale cereale α*BIII‐rye *α*‐amylase inhibitor	Transgenic overexpression	*Nicotiana tabacum*	*Anthonomus grandis*	cDNA overexpression was driven by a phytohemagglutinin promoter. Transgenic seeds accumulated from 0.1% to .028% of seed protein	Bioassays using transgenic seed flour mixture for an artificial diet resulted in 74% mortality in first instar larvae of *A. grandis*	[118]

^a^ GenBank (NCBI) accession n. J01261 and U10348; ^$^ AK107328.

**TABLE 3:** Granted and active patents of plant‐derived *α*‐amylase inhibitors for insect pest control.

Number	Application	Granted	Status	Country	Applicant	Title	Description	Target
390224	2015‐09‐25	2022‐02‐24	Active	India	Rajalakshmi Engineering College	Guava leaf extract stored food grain insecticide	*Psidium guajava* leaf extract and use as an *α*‐amylase inhibitor‐related biopesticide against insect pests in stored food grains	*Sitophilus oryzae* and *Tribolium castaneum*
US9567381B2 JP6308955B2	2013‐03‐08	2017‐02‐14 2018‐04‐11	Active	USA Japan	Vestaron Corporation	Toxic peptide production, peptide expression in plants, and combinations of cysteine‐rich peptides	Cysteine‐rich insecticidal peptides derived from *Bacillus thuringiensis* and their genes and endotoxins in combination with toxic peptides known as *ICK* genes	Insect pests
US10941387B2 BRPI1102841B1	2012‐06‐08	2021‐03‐09	Active	USA Brazil	Brazilian Agricultural Research Corporation	*α*‐Amylase mutant inhibitors isolated from *Phaseolus vulgaris* with properties of controlling insect pests, compositions containing such mutants, and method of using thereof	The invention provides new *α*AIs analogous mutant molecules for controlling insect pests, in particular, *Anthonomus grandis*	*Anthonomus grandis*
BRPI1107433B1	2011‐12‐30	2021‐01‐26	Active	Brazil	Brazilian Agricultural Research Corporation	Computational design for new *α*‐amylase inhibitors	Method for performing the computational design of new compounds with potential inhibitory function of the *α*‐amylase enzymes produced by insects that primarily feed on stored agricultural products	Insect pests

The biological activity of these inhibitors, when overexpressed in transgenic plants against *α*‐amylases of insect pests or non‐target organisms, has been extensively studied, indicating that genetic engineering can replicate the natural inhibitory activity observed in the original plant [24]. Notably, overexpression driven by constitutive and seed‐specific promoters, such as the CaMV 35S and *Phaseolus vulgaris* phytohemagglutinin promoters, results in a high accumulation of inhibitor protein in transgenic plants or seeds [8]. For example, the transgenic overexpression of the *αAI* gene driven by a strong seed‐specific promoter improved seed resistance to pea weevil, cowpea and azuki bean weevils, and coffee berry borer in the lab and in the field (Table 2) [18, 19, 20, 21, 23, 24, 27, 44]. However, before any of these promising candidate genes can be deployed commercially in food crops, biosecurity regulators will consider whether they might be toxic, anti‐nutritional, or allergenic for humans, and whether they might be toxic to non‐target organisms [121, 122, 123, 124, 125, 126, 127, 128, 129, 130]. Therefore, selecting or optimizing *α*‐amylase inhibitor molecules that specifically target enzymes in insect pests, while minimizing activity against enzymes in non‐target organisms such as humans and animals, is crucial for developing safe and effective plant protection strategies [14, 26, 43, 131, 132]. In addition, editing endogenous genes encoding *α*‐amylase inhibitors using CRISPR/Cas9 may be a strategy to reduce their anti‐nutritional effects, not necessarily by knocking out the genes, but by deleting specific protein regions associated with allergenic impacts on humans or animals [133, 134]. Additionally, modulating the promoter of these endogenous genes through CRISPR‐mediated transcriptional activation (CRISPRa) or editing *cis*‐acting regulatory elements could enhance transcriptional activity or responsiveness via CRISPR/Cas9 homology‐dependent repair (HDR) [135]. Furthermore, stacking genes encoding *α*‐amylase inhibitors may be a strategy to improve the efficacy of these molecules and enhance the durability of transgenic plants technology against insect pests.

Finally, the growing demand for environmentally friendly technologies could be aligned with *α*‐amylase inhibitor‐based biotechnological tools, given their environmental safety, target specificity, and practical applicability compared to existing pest control methods, as well as their potential to reduce the reliance on non‐selective agrochemicals [7, 136, 137]. Therefore, prior research, careful planning, and engineering of these molecules are essential to maximize their inhibitory activity in crops against insect pests, prevent impacts on non‐target organisms, and minimize the risk of resistance development.

## 7 Concluding Remarks

Insect pests possess *α*‐amylases that facilitate the breakdown of starch from parasitized plants or seeds, allowing them to access sugars for sustenance. In response, plants develop defense mechanisms, notably *α*‐amylase inhibitors, to counteract exogenous *α*‐amylases, particularly those from insect pests. Conversely, insect pests have developed resistance or tolerance mechanisms to these plant‐derived inhibitors. Cultivated cereal and legume grains are frequently susceptible to insect pest attacks, especially during storage. Engineering crops to enhance resistance through the transgenic overexpression of genes encoding *α*‐amylase inhibitors presents a promising biotechnological solution. Numerous plant genes encoding *α*‐amylase inhibitors that have been transferred into crops have demonstrated significant increases in plant resistance against target insect pests, both in the laboratory and occasionally in the field. However, there is often a reluctance to accept transgenic crops in some communities, which has hindered the widespread adoption of insect pest control technologies based on inhibitors. Nevertheless, field trials with transgenic plants overexpressing *α*‐amylase inhibitors have been conducted, representing an important step toward the commercialization of crops carrying these genes. The integration of advanced biotechnological approaches into inhibitor‐enzyme interaction studies offers significant potential, enabling the design and discovery of new molecules with optimized properties. Techniques such as structural biology, high‐throughput screening, and molecular modeling can accelerate the identification of highly selective *α*‐amylase inhibitors. These approaches provide insights into innovative insect pest control solutions that are both effective and safer for non‐target species, thereby supporting sustainable agricultural practices.

## Author Contributions

**Marcos Fernando Basso**: conceptualization, writing–review and editing, writing–original draft, data curation, investigation. **Arnubio Valencia‐Jiménez**: writing–review and editing, investigation. **Fabrizio Lo Celso**: investigation, writing–review and editing, formal analysis. **Isabel Rodrigues Gerhardt**: investigation, writing–review and editing. **Thomas Joseph V. Higgins**: investigation, writing–review and editing, conceptualization. **Maria Fatima Grossi‐de‐Sa**: conceptualization, funding acquisition, investigation, writing–review and editing, supervision.

## Ethics Statement

The authors have nothing to report.

## Consent

The authors have nothing to report.

## Conflicts of Interest

The authors declare no conflicts of interest.

## Supporting information




**File S1**. Plant‐derived *α*‐amylase inhibitor protein sequences of the knottin family.

**File S2**. Plant‐derived *α*‐amylase inhibitor protein sequences of the thionin family.

**File S3**. Plant‐derived *α*‐amylase inhibitor protein sequences of the CM‐protein family.

**File S4**. Plant‐derived *α*‐amylase inhibitor protein sequences of the kunitz‐type family.

**File S5**. Plant‐derived *α*‐amylase inhibitor protein sequences of the thaumatin family.

**File S6**. Plant‐derived *α*‐amylase inhibitor protein sequences of the lectin‐like family.

**File S7**. Actinobacteria‐derived *α*‐amylase inhibitor protein sequences of the *Streptomyces* sp. family.

**File S8**. The *α*‐amylase inhibitor protein sequences of seven families used for phylogeny analysis.

**File S9**. The *α*‐amylase inhibitor protein sequences of seven families used for MEME motif analysis.

## Data Availability

Data sharing is not applicable to this article as no new data were created or analyzed in this study.
